# Synthesis, Spectroscopic, and Theoretical Study of Copper and Cobalt Complexes with Dacarbazine

**DOI:** 10.3390/ma14123274

**Published:** 2021-06-13

**Authors:** Grzegorz Świderski, Ryszard Łaźny, Michał Sienkiewicz, Monika Kalinowska, Renata Świsłocka, Ali Osman Acar, Aleksandra Golonko, Marzena Matejczyk, Włodzimierz Lewandowski

**Affiliations:** 1 Department of Chemistry, Biology and Biotechnology, Bialystok University of Technology, Wiejska Street 45E, 15-351 Bialystok, Poland; m.kalinowska@pb.edu.pl (M.K.); r.swislocka@pb.edu.pl (R.Ś.); m.matejczyk@pb.edu.pl (M.M.); 2 Faculty of Chemistry, University of Bialystok, Ciolkowskiego Street 1K, 15-245 Bialystok, Poland; lazny@uwb.edu.pl (R.Ł.); mikes@uwb.edu.pl (M.S.); 3 Material Science and Nanotechnology Engineering, TOBB University of Economics and Technology, 06-560 Ankara, Turkey; aacar@etu.edu.tr; 4 Institute of Agricultural and Food Biotechnology, Department of Microbiology, Rakowiecka 36, 02-532 Warsaw, Poland; olau.95@gmail.com

**Keywords:** dacarbazine, triazene, anticancer properties, 3d metal complexes, spectroscopy (IR, Raman), NMR

## Abstract

Dacarbazine (DAC) 5-(3,3-dimethyl-1-triazenyl)imidazole-4-carboxamide is an imidazole-carboxamide derivative that is structurally related to purines. DAC belongs to the triazene compounds, which are a group of alkylating agents with antitumor and mutagenic properties. DAC is a non-cell cycle specific drug, active in all phases of the cellular cycle. In the frame of this work the 3d metal complexes (cobalt and copper) with dacarbazine were synthesized. Their spectroscopic properties by the use of FT-IR, FT-Raman, and ^1^HNMR were studied. The structures of dacarbazine and its complexes with copper(II) and cobalt(II) were calculated using DFT methods. The effect of metals on the electronic charge distribution of dacarbazine was discussed on the basis of calculated NBO atomic charges. The reactivity of metal complexes in relation to ligand alone was estimated on the basis of calculated energy of HOMO and LUMO orbitals. The aromaticity of the imidazole ring in dacarbazine and the complexes were compared (on the basis of calculated geometric indices of aromaticity). Thermal stability of the investigated 3d-metal complexes with dacarbazine and the products of their thermal decomposition were analyzed.

## 1. Introduction

Dacarbazine (DAC) [5-(3,3-Dimethyl-1-triazenyl) imidazole-4-carboxamide] belongs to the alkylating agents of triazene group use in anticancer therapy [1,2,3,4]. DAC is an imidazole-carboxamide derivative that is structurally related to purines. DAC is a non-cell cycle specific drug, active in all phases of the cellular cycle. This drug is used for chemotherapy in different types of cancer malignant melanoma, soft-tissue sarcoma, osteogenic sarcoma, neuroblastomas, and Hodgkin’s disease [5,6]. The detailed molecular mechanism of DAC activity is unclear. Research to date indicates three possible mechanisms of action of this drug: (a) inhibition of DNA synthesis by acting as a purine analog, (b) action as alkylating agent, and (c) interaction with SH groups [7]. Hayward et al. [8], proposed another mechanism of DAC activity based on antimetabolite-like activity. In this mechanism, DAC plays the role of an inhibitor of purinic base incorporation into DNA during DNA synthesis. However, this mechanism does not seem to play a key role in the antitumor activity of DAC [8].

**Figure 1:**
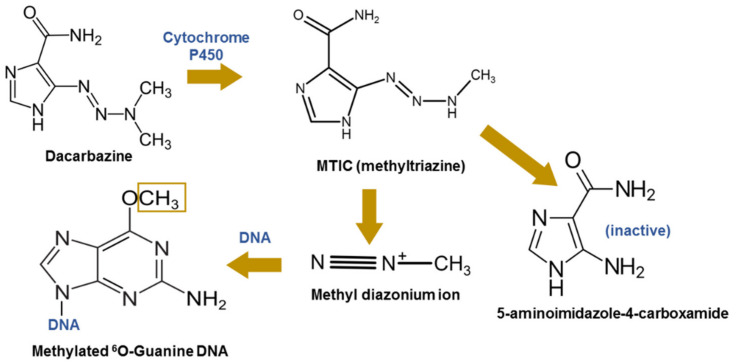
Mechanism of action of dacarbazine.

Based on experimental data it seems that alkylation of nucleic acid is the primary mode of antitumor DAC action [7]. DAC as an alkylator donates methyl groups to many sites in DNA. A very common place of attack of DAC is the O(6)-position of guanine resulting in O(6)-methylguanine (O(6)-MeG) which frequently mispairs with thymine during DNA duplication. Each cell is equipped with repair systems, the mismatch repair (MMR) system recognizes O(6)-MeG:T mismatches which lead to cell cycle arrest and cell death. If MMR is defective, the cell will divide and GC → AT transition mutations occur (Figure 1) [5,6]. Generally, DAC causes DNA damage, cell cycle arrest and apoptosis [9]. Hayward et al. [8] proposed another mechanism of DAC activity based on antimetabolite-like activity. In this mechanism, DAC plays the role of an inhibitor of purinic base incorporation into DNA during DNA synthesis. However, this mechanism does not seem to play a key role in the antitumor activity of DAC [8]. DAC is a prodrug that is activated by N-demethylation in liver microsomes with participation of the liver’s microsomal enzymes (CYP1A1, CYP1A2, and CYP2E1) that hydroxilate and N-demethylate the DAC [7].

Studies by earlier authors have shown that DAC, in combination with other drugs is used for treating renal adenocarcinoma, soft-tissue sarcoma and malignant lymphomas. Al-Qatati and Aliwaini [9] in their work, presented that combined pitavastatin with DAC treatment induces cell death through intrinsic apoptosis in melanoma cells. Naserian et al. [10] investigated the cytotoxicity and apoptosis of the metformin alone and in combination with DAC in Raji and Ramos lymphoma cell lines. Metformin showed synergistic cytotoxic effects in combination with DAC, reduced cell viability, and increased apoptosis in Raji and Ramos lymphoma cells in comparison with the use of each drug alone. Additionally, Finotello et al. [11] reported that results of in vitro and in vivo studies suggest that dacarbazine acts synergistically with anthracyclines and has a moderate effect on the treatment of high-grade sarcomas in humans.

The greatest obstacle in the treatment of patients with advanced stages of melanoma and other cancers is the unique tumor resistance, both primary and secondary, to all known compounds including dacarbazine. The complexation of metal ions with anticancer drugs creates the possibility of an increase in the bioavailability and activity of drugs [12,13]. Metal complexes play a very important role in modern cancer therapy. The complexation of metal ions with biologically active ligands can change their therapeutic activity. Metal ions affect the distribution of the electronic system of ligands which in turn changes the reactivity and biological activity of the ligands [14,15]. Complexes with some of the transition metals, e.g., Au, Ag, Co, Cu, Ni, Fe Pt(II) and (IV), Pd(II), and Ru(III) show high anticancer activity [16].

DAC possesses a triazene group connected to the imidazole ring which is attached to the carboxamide group. In the structure of the DAC, they are atoms that may be donors of electron pairs, making complexation of the ligand with metal ions possible. Different types of metal-ligand complexes can be formed when the molecule of dacarbazine coordinates metal through seven potential donor atoms. 

Several studies have shown that complexation of dacarbazine with metal ions may change the reactivity and stability of the molecule, thereby improving its therapeutic properties. In vitro and in vivo studies have shown that the iron(III) complex of dacarbazine may exhibit an increased antitumor effect compared to the ligand alone [17,18]. Temerk and Ibrahim [19] in their work investigated the influence of DAC complexation with Cu(II) on the drug intercalation to DNA. The results of that experiment showed that the interaction of DAC with dsDNA in the presence of Cu(II) led to a much stronger intercalation, compared to DAC alone [19]. 

Our studies showed that there is a dependency between the electronic charge distribution in metal complex and salt molecules and the location of these metals in the periodic table [20]. Alkali metal cations and some heavy metal cations (Ag(I), Pb(II), Hg(I), Hg(II)) perturb the electronic system of ligands (benzoic, salicylic, pyridinecarboxylic acids and others), whereas 3d and 4f metal cations stabilize it [21,22,23,24,25,26]. This conclusion allows us to foresee the changes in the electronic structure under the influence of metal cations and estimation of the physicochemical and biological properties of complexes. Thermal analysis allows us to determine the thermal stability of the investigated metal complexes and to determine the residues of this process. We have repeatedly tested the thermal stability of ligands of high biological importance and their complexes with metals. We found that metal oxides are the final product of the decomposition of metal complexes under the oxygen conditions of the process, with the use of a sufficiently high temperature [27,28,29,30,31]. This may be of practical importance when thermal disposal of drugs based on metal complexes with particularly toxic ligands, such as dacarbazine, is used.

In the frame of this work the 3d metal complexes with dacarbazine were synthesized. The aim of the work was to estimate the effect of metal ions (copper(II) and cobalt(II)) on the reactivity and electronic stability of dacarbazine. For this purpose, the spectroscopic properties of compounds were studied by the use of FT-IR, FT-Raman, and ^1^HNMR. Thermal stability of the investigated 3d-metal complexes (cobalt(II) and copper(II)) with dacarbazine and the products of their thermal decomposition were analyzed. 

Synthesis of complexes with metals such as copper and cobalt is a noticeable trend in many fields of science, such as pharmaceuticals and technologies related to catalysis and sensors. Padnya et al., with high efficiency, synthesized a series of catechol-containing Schiff bases, tetrasubstituted on the lower rim thiacalix [32] arene derivatives in three stereoisomeric forms, cone, partial cone and 1,3-alternate, which can be used as a component of antimicrobial agents, catalysts, chemical, and electrochemical sensors [32]. On the other hand, the copper complexes described by Colombo et al. can be applied as eco-sustainable redox mediators in sye-sensitized solar cells [33].

In the case of compounds with potential biological applications, it should be noted that some metal complexes with many oxidation states, e.g., vanadium, undergo many chemical transformations under physiological conditions. So the study of the mechanism of action should assume that the complexes do not exist in cells/tissues in its original form. The authors of the review suggest the necessity of speciation of the vanadium species formed in each biological compartment and defining the defined reaction conditions under which the complex compound acts [34]. This is especially important when assessing the interaction of such complexes with biological macromolecules, e.g., proteins or DNA. Currently, there are many inorganic coordination compounds used as antibiotics (bleomycin), in the treatment of neoplastic diseases (cisplatin) or anti-arthritis drugs (auranofin) [35]. Metal complexes also offer hope for a more effective treatment of solid tumors in which there is a hypoxic state with a more acidic and reducing environment. Studies on compounds containing Pt^4+^, Ru^3+^, and Co^3+^ have shown the potential for selective action in cancer cells. Cobalt, unlike ruthenium or platinum, is non-toxic and, when reduced from Co^3+^ to Co^2+^ under hypoxic conditions, the complex becomes labile, which enables drug dissociation. In experimental research, de Souza et al. proposed cobalt(III) L-Phe complexes as a model for the hypoxia-activated delivery of melphalan [36].

## 2. Materials and Methods

### 2.1. Synthesis

Complexes dacarbazine-CoCl_2_ (DAC)_2_*1.75CH_3_OH and CuCl_2_(DAC)_2_*1.5CH_3_OH were obtained by the same method of synthesis by reaction dacarbazine of metal chlorides in a methanolic medium. The weight amount of dacarbazine (0.2 mol) was dissolved in cold methanol and it was added to 5 mL of methanolic solution of metal chloride (0.1 mol) (CuCl_2_, CoCl_2_). Then the mixture was shaken for 2 h in the water bath in room temperature. Next, 50 mL of diethyl ether was added and it was left for 2 h. The precipitate was washed three times with 50 mL of diethyl ether. The obtained complexes were dried in a vacuum for 72 h.

Elemental analysis: Anal. Calc. for CoCl_2_(DAC)_2_*1.75CH_3_OH: C, 29.62; H, 4.75; N, 30.72. Found: C, 29.35; H, 5.03; N, 30.318. Calc. for CuCl_2_(DAC)_2_*1.5CH_3_OH: C, 29.98; H, 4.91; N, 30.53. Found: C, 30.13; H, 5.30; N, 30.82.

### 2.2. Methods

The FT-IR spectra were recorded with an Alfa (Bruker) spectrometer (Billerica, MA, USA) within the range of 400–4000 cm^−1^. Samples in the solid state were measured in KBr matrix pellets and ATR technique. FT-Raman spectra of solid samples were recorded in the range of 100–4000 cm^−1^ with a MultiRam (Bruker) spectrometer (Billerica, MA, USA). The resolution of the spectrometer was 1 cm^−1^. The ^1^H spectra of DMSO solution of studied compounds were recorded with a Bruker Avance II 400 MHz (Billerica, MA, USA) unit at room temperature (sample content 5 mg/1 mL of solvent). TMS was used as an internal reference. Elemental analysis for the weight percentages of carbon and hydrogen was done with Perkin-Elmer 240 (Waltham, MA, USA) equipment. To calculate optimized geometrical structures, NMR and IR spectra, NBO analysis, HOMO/LUMO orbitals of dacarbazine, and complexes with copper and cobalt were used with the density functional (DFT) hybrid method B3LYP with nonlocal correlation provided by the Lee–Young–Parr expression. All calculations were carried out with 6-311++G(d,p) basis set and performed with GAUSSIAN 09 [37] packed.

The aromaticity indices (HOMA, GEO, EN, I6) were calculated for geometric structures (theoretical and calculated) of dacarbazine and its complexes. The HOMA index (harmonic oscillator model of aromaticity) differs from all other geometry-based ones by assuming another reference bond length. In this model, instead of the mean bond length a concept of the optimal bond length is applied [38]:

$$HOMA=1-[\alpha {\left(R_{opt}-R_{av}\right)}^{2}+\frac{\alpha }{n}\sum {\left(R_{av}-R_{i}\right)}^{2}]=1-EN-GEO$$

Within the confines of the HOMA model, it is possible to obtain two components which describe different contributions to decrease in aromaticity, i.e., (a) due to bond elongation (the EN component), and (b) due to bond length alternation (the GEO component). The value of HOMA index is equal to 1 for the entire aromatic system; HOMA = 0 when the structure is non-aromatic and HOMA < 0 for an anti-aromatic ring.

The value of the Bird’s aromaticity index (I_5_, I_6_) describes the equation [39]:

$$I=100\left\{1-\left(\frac{V}{V_{k}}\right)\right\}$$

where: *V_k_* is for the five-membered rings 35 and the six-membered 33.3, and *V* is calculated from the equation:

$$V=\left(\frac{100}{n_{av}}\right){\left[\overset{n}{\underset{r=1}{\sum }}{\left(n_{r}-n_{av}\right)}^{2}/n\right]}^{1/2}$$

where: *n_av_*—average binding order, *n*—bond order based on bond length: *n* = (*a*/R) − b, *a* and b—parameters depending on the type of atoms in the bond.

NBO analysis was performed for the optimized structures to determine the electronic charge distribution [40]. Calculations were made using the B3LYP/6-311++G(d, p) method.

Thermal analyses of the prepared complexes were performed by the thermogravimetric (TG) methods using the Perkin-Elmer analyzer in a dynamic air atmosphere. Therefore, 6.0–8.0 mg samples were heated in the range of 50–890 °C in the ceramic crucibles using a heating rate of 10 °C min^−1^. The products of dehydration and decomposition processes were determined from the TG curves.

The crystal structure of the DNA dodecamer was obtained from Protein Database (PDB ID: 1BDNA) and used for molecular modeling studies carried out in AutoDock 4.2 MGL Tools package supplied with AutoGrid4.0 and AutoDock4.0 [41]. In order to analyze the obtained results and compare the number of hydrogen bonds formed, the VMD (Visual Molecular Dynamics) program was used [42]. 

AutoGrid4.0 was used to compute grid maps using a grid box. The molecular docking was carried out by setting the grid box size to cover the predicted binding sites, using 52, 56, 118  x, y, z points with a grid spacing of 0.375 Å. The grid center was set to 14.72, 21.006, 8.801 x, y, and z dimensions, respectively. The Lamarckian genetic algorithm (LGA) was selected to generate the best ligand conformers. We successfully performed 1000 docking runs with AutoDock4.0 for each ligand (DAC1, DAC2) and Co(II)-DAC, Cu(II)-DAC complexes. As standard values for hydrogen bond formation assumed distance 2.9 Å and cutoff angle 60 degrees (120 to 180 degrees). To visualize results BIOVIA Discovery Studio and USCF Chimera 1.10.2 were used [43,44]. 

## 3. Results and Discussion 

### 3.1. IR and Raman Spectra

**Figure 2:**
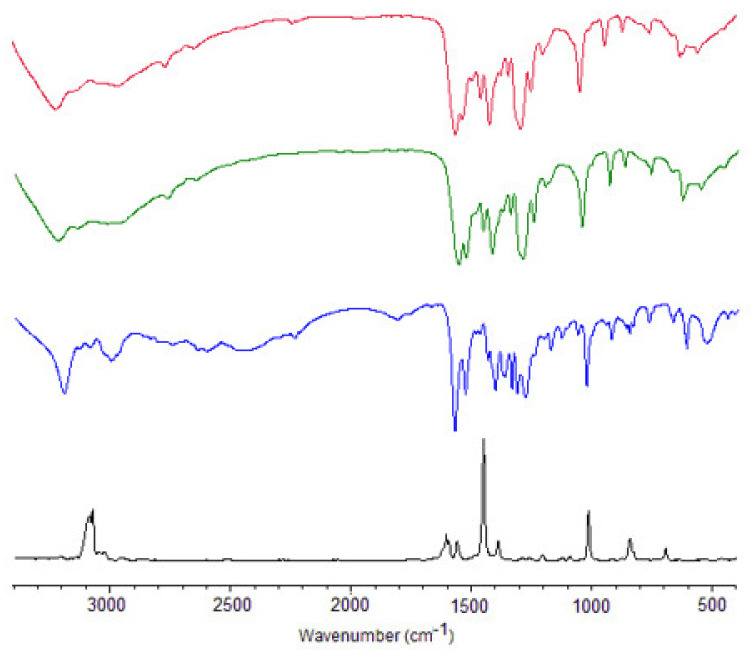
IR spectra of dacarbazine (blue line), cobalt complex (red line), copper complex (green line), and Raman spectra of dacarbazine (black line).

The IR and Raman spectra of dacarbazine and IR spectra of cobalt(II) and copper(II) complexes of dacarbazine are shown in Figure 2.

**Figure 3:**
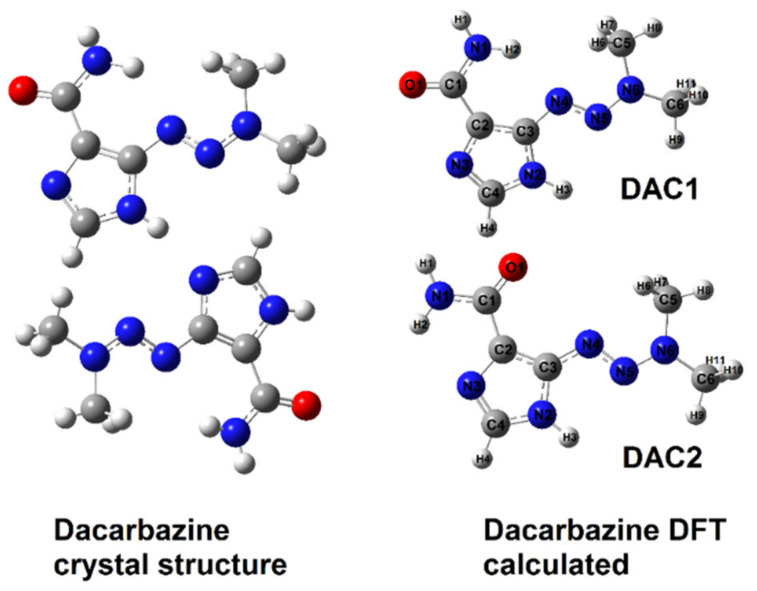
Crystal and theoretical structure (B3LYP/6-311++g(d,p)) of dacarbazine.

The wavenumbers and intensities of selected bands from the experimental and theoretical IR and Raman spectra of ligand and experimental IR spectra of complexes are gathered in Table 1. The assignment of bands was done on the basis of theoretical wavenumbers obtained at the B3LYP/6-311++G** level as well as literature data [45]. The theoretical wavenumbers were scaled to reproduce adequately the experimental wavenumbers (f = 0.967). Some characteristic bands occurred in the experimental spectra of dacarbazine, i.e., bands assigned to the stretching vibrations of the carbonyl group νC=O at 1609 cm^−1^ (IR) and 1604 cm^−1^ (Raman). The theoretical wavenumbers of the νC=O band were 1691 cm^−1^ (for the DAC1 conformer) and 1683 cm^−1^ (in the case of the DAC2 conformer) (Figure 3) (the values scaled by the factor f = 0.967). In the IR spectra of Co(II) and Cu(II) complexes of dacarbazine the νC=O band was of similar wavenumbers and reduced intensities compared with the spectra of ligand (i.e., 1605 cm^−1^—Co(II) complex and 1609 cm^−1^—Cu(II) complex). This suggested that the carbonyl group did participate in metal ion coordination. In the spectra of dacarbazine and its metal complexes, the characteristic bands assigned to the vibrations of NH_2_ (of amide group) occurred. There were bands of the stretching asymmetric vibrations ν_as_NH_2_ located at 3383 cm^−1^ (IR) and 3371 cm^−1^ (Raman) as well as stretching symmetric vibration ν_s_NH_2_ near 3269 cm^−1^ (IR). In the spectra of complexes, the bands assigned to the stretching vibrations ν_as_NH_2_ were significantly shifted whereas bands of ν_as_NH_2_ disappeared compared with the appropriate bands in the spectra of ligand. Significant changes were also observed in the case of deforming out-of-plane vibrations of NH_2_ group (ρNH_2_), i.e., 1436 cm^−1^ in the spectrum of ligand, 1440 cm^−1^ for Cu and Co complexes as well as 542 cm^−1^ for ligand and 565 cm^−1^ for the Co complex and 568 cm^−1^ for the Cu complex. The metal complexation affected the location of the band derived from the vibrations of the C-NH_2_ group as well. Namely, the band of deforming out-of-plane vibrations ρC-NH_2_ was located at 1476 cm^−1^ and underwent movement to the 1487 cm^−1^ and 1488 cm^−1^ in the cobalt and copper complexes of dacarbazine, respectively. The characteristic changes in the wavenumbers of the bands assigned to the vibrations of N=N–N and CH_3_ groups were observed.

The band assigned to the deformations of the triazene group αNNN was located at 630 cm^−1^ in the spectra of ligand, and then was shifted toward higher wavenumbers in the spectra of complexes (649 cm^−1^—Cu complex and 646 cm^−1^—Co complex). The symmetric stretching vibrations of the methyl group ν_s_CH_3_, which were present in the spectra of ligand at 3147, 2946, 2753, and 2612 cm^−1^ disappeared in the spectra of metal complexes. Whereas band of asymmetric stretching vibrations of the methyl group ν_s_CH_3_ were significantly shifted in the spectra of complexes compared with the spectra of ligand, i.e., 2905 cm^−1^ in the IR spectra of dacarbazine and 2925 cm^−1^ and 2923 cm^−1^ in the IR spectra of Co and Cu complexes, respectively. The band of the deforming in-plane vibrations of the methyl group of dacarbazine disappeared or decreased/increased in their wavenumbers in the spectra of Cu and Co complexes (Table 1). 

Moreover, the coordination of copper(II) and cobalt(II) by dacarbazine caused changes in the spectra of complexes in the region of bands assigned to the stretching (ν_ring_) and deforming (ring_def_) vibrations of the ring (compared with the appropriate bands in the spectra of dacarbazine). Namely, ν_ring_ bands located at 1381, 1270, 1231 cm^−1^, and ring_def_ bands near 882 and 450 cm^−1^ disappeared in the spectra of metal complexes. Whereas ν_ring_ bands situated in the spectra of ligand at 1344 and 1402 cm^−1^ were slightly shifted to the higher wavenumbers in the spectra of complexes. The band of stretching vibrations of the CH bond of the aromatic ring of dacarbazine located at 3174 cm^−1^ was shifted toward higher wavenumbers in the spectra of complexes (i.e., 3188 cm^−1^—Co(II) complex and 1386 cm^−1^—Cu(II) complex). The deforming vibrations of the CH bond of the ring slightly moved to the higher wavenumbers in the spectra of complexes compared with the spectra of ligand.

The difference between the location of the appropriate bands in the spectra of dacarbazine and copper(II) and cobalt(II) complexes of dacarbazine might be caused by the coordination of metal ions by the oxygen atom of carbonyl group and nitrogen atom of imidazole ring. 

### 3.2. NMR Spectra

In the experimental ^1^HNMR spectra of dacarbazine, four groups signals from protons were observed (Table 2 and Figure S1). The values of the chemical shift for two protons of the amide group have been values 7.41 and 7.29 ppm. This signal possessed very low intensity in the spectra of the cobalt complex, and was shifted to 6.20, 5.40, giving extended signals, due to changes in the electronic density in the amide group, due to the attachment of a metal to this group. In the spectra of ligand, the signals at 3.13–3.50 ppm were assigned to the protons of the methyl group.

Complexation of dacarbazine by the cobalt caused insignificant displacement of this signal. On the basis of the value of signals deriving from the aromatic protons (imidazole ring), the effect of metal ions on the electronic charge distribution of ligand might be discussed. The movement of the chemical shifts of aromatic protons toward higher wavenumbers showed an increase in the aromaticity of the ring as well as the stabilization of the aromatic system [20]. In the case of cobalt(II) complexes of dacarbazine, an increase in the value of the chemical shift of protons of CH bond of the aromatic ring was observed (Figure S1). Proton NMR spectrum for the copper complex could not be recorded. The theoretically calculated chemical shifts in the proton spectrum of the copper complex show similar trends in proton shifts relative to the ligand as in the cobalt complex. Cobalt and copper possess a stabilizing effect on the electronic system of ligand. The same effect was observed on the basis of the IR spectra of Co and Cu complexes of dacarbazine (an increase in the values of the wavenumbers and intensities of the aromatic ring). Our long-term study on the influence of metals on the electronic system of ligand (aromatic and heteroaromatic system of five- and six-membered carboxylic acids) showed that 3d transition metals (including cobalt and copper) stabilize the electronic system of ligands [20,21,22,23,24]. In the spectra of such complexes (a) an increase in the values of signals of the aromatic protons (in ^1^HNMR spectra), and (b) an increase in wavenumbers and intensity of bands assigned to the aromatic system (in the IR spectra) compared with the appropriate signals/bands in the spectra of ligand were observed.

### 3.3. Structure, Aromaticity, and NBO Analysis of Dacarbazine and Copper and Cobalt Complexes

**Figure 4:**
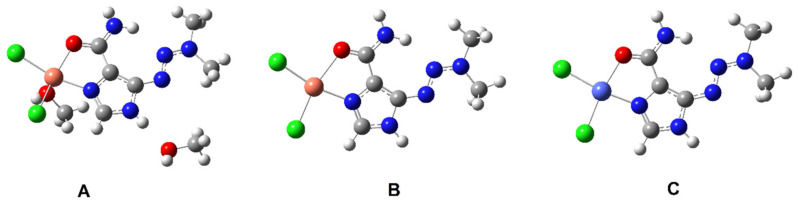
Crystal (**A**), theoretical structure (B3LYP/6-311++g(d,p)) of cobalt (**B**) and copper (**C**) complex with dacarbazine.

The dacarbazine molecule has a triazene moiety linked to an imidazole ring. IR, NMR studies and literature X-ray data on the structure of dacarbazine complexes [46] showed that metals such as copper and cobalt coordinate the ligand (dacarbazine) through the carbonyl group and the nitrogen atom from the imidazole ring. A similar type of metal-ligand coordination has been observed for 3d-transition metal complexes with imidazole carboxylic acid [38]. On the basis of spectroscopic studies (IR, Raman) and theoretical calculations, it was found that 3d metals affect the electronic charge distribution of the aromatic ring of ligand. With the increase in the electronic stability of the ligand, the reactivity of the compound changes, which is important in the case of biologically active ligands. Theoretical structures of dacarbazine complexes with cobalt and copper were calculated. The calculated bond lengths and the values of the selected angles were compared with these ones obtained for experimentally solved structures (X-ray) available in the literature. The geometric indices of aromaticity (HOMA, GEO, EN, and I5) and the electronic charge distribution by the NBO method were also calculated. Figure 3 shows the experimentally determined structure [46] and the calculated two dacarbazine conformers (at B3LYP method). Figure 4 shows the experimental structure of the complex of copper with dacarbazine (A) [47] and the theoretically modeled structures of the complexes with copper (B) and cobalt (C).

Both theoretically modeled dacarbazine conformational structures have similar energy values. According to the calculated geometric aromaticity indices, the imidazole ring of the DAC1 structure is more stable than the DAC2 structure (Table 3). The system is stabilized by an intramolecular hydrogen bond between the hydrogen atom of the amino group and the nitrogen atom of the triazene group. The length of this bond is 2.154 Å. In the DAC2 structure, the amide moiety is rotated 180° along the C1–C2 carbons as compared to the DAC1 structure. The aromatic system of imidazole in this structure is less stable, as evidenced by the values of the aromaticity indices. The structure of the DAC1 monomer is the same as that of experimentally determined dacarbazine. In the structure determined by the X-ray diffraction method [42], there is an intramolecular hydrogen bond that stabilizes the aromatic system between the hydrogen atom of the amino group and the triazene nitrogen atom with a length of 2.294 Å, and an intermolecular hydrogen bond with a length of 1.945 Å. The values of geometric aromaticity indices calculated for the experimental structure and the theoretically modeled DAC1 are at a similar level (Table 3). The differences in the lengths of the corresponding bonds in the experimentally determined dacarbazine structure and the theoretically modeled DAC1 monomer range from 0.001 to 0.026 Å.

The complexation of dacarbazine with 3d transition metals (copper and cobalt) influences the stability of the ligand’s electronic system, which is observed by changing the aromaticity of the system expressed in the values of aromaticity indices, changes in the distribution of electronic charge, and changes in the bond lengths in the studied systems. The comparison of the structure of the dacarbazine complex with copper [47] to dacarbazine [46] shows that the aromatic system of the imidazole ring is stabilized by complexation (Table 3). It was observed both when comparing the ligand structure to the complex for the experimental and theoretically modeled structures (the effect of the metal is more noticeable when comparing the experimental structures than the theoretically modeled ones). Under the influence of copper, there is a significant change in the length of the imidazole ring bonds, which results in an increase in the HOMA and I5 indices. Complexation of metals by ligand slightly changes the bond length of the triazene moiety (Table 3). The complexation of cobalt with dacarbazine influences the change of aromaticity of the imidazole ligand ring to a lesser extent than complexation of copper (when comparing theoretical structures). A slight increase in the value of the I5 index and a slight decrease in the value of the HOMA index were observed. The theoretical structures of copper and cobalt complexes have similar bond lengths and angles.

It was observed that the complexation of dacarbazine with copper and cobalt slightly changed the bond length of the N4-N5-N6 triazene moiety and the length of the N6-C5 and N6-C6 bonds (nitrogen N6 with methyl groups). The analysis of the NBO electronic charge distribution (Table 4) showed that the values of the electronic charges on the nitrogen atoms of the triazene group and the carbons of the methyl groups are similar in the complexes and the ligand. 

On the other hand, the influence of metals on the distribution of electronic charge on the imidazole ring of dacarbazine was observed. The electronic density around the carbon atom labeled C2 and the nitrogen atom N3 increased in copper and cobalt complexes compared to the ligand, while around the C3, C4, and N2 atoms the electronic density decreased (Table 4). The effect of copper on the electronic charge distribution in the imidazole ring of the ligand is greater than that of cobalt. The total electronic density of the C-ring of the copper complex is greater than that of the cobalt complex. Studies of 4-imidazole carboxylic acid complexes with 3d-transition metals (including copper and cobalt) also showed that copper and cobalt had a stabilizing effect on the aromatic system of the imidazole ring [48].

### 3.4. HOMO and LUMO Orbitals

**Figure 5:**
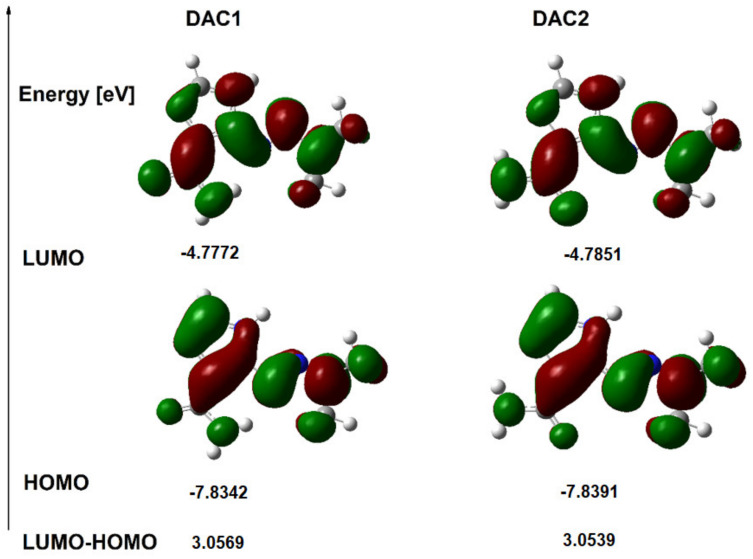
HOMO and LUMO orbitals of dacarbazine.

**Figure 6:**
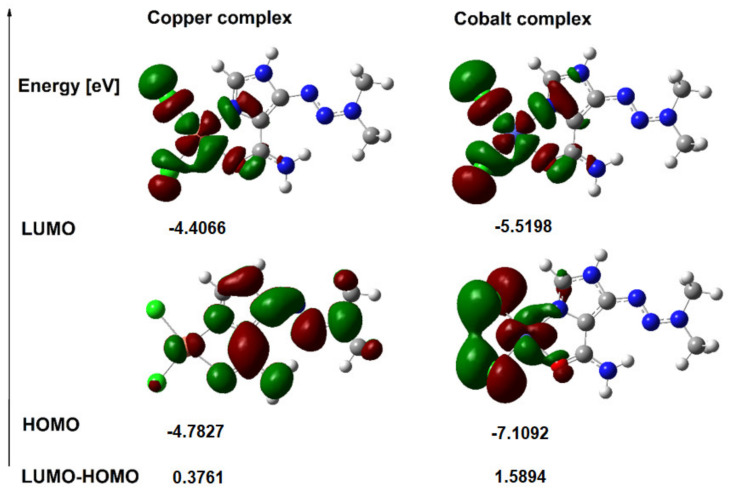
HOMO and LUMO orbitals of copper and cobalt complexes with dacarbazine.

The energy values of the HOMO and LUMO orbitals for dacarbazine as well as copper and cobalt complexes were calculated. The shapes of the orbitals are shown in Figure 5 and Figure 6, while in Table 5 the values of the energy of the orbitals and other electronic parameters are gathered. The highest-occupied molecular orbital (HOMO) and the lowest unoccupied molecular orbital (LUMO) play an important role in predicting charge transfer in the molecule, chemical reactivity, bioactivity, and compound stability [46]. The shapes of the LUMO and HOMO molecular orbitals of the dacarbazine complexes with copper and cobalt are shown in Figure 6. The energy values of the HOMO and LUMO orbitals and the value of the energy difference between the HOMO-LUMO levels for the studied compounds are presented in Table 5. The value of the HOMO-LUMO energy difference (Energy gap) for the copper complex is 0.3761 eV, and for the cobalt complex it is 1.5894 eV and the values are lower than those obtained for dacarbazine (3.057 eV for DAC1). This indicates a decrease in kinetic stability and an increase in the reactivity of the complexes toward the ligand. Other general reactivity descriptors [49,50] such as ionization potential (I), electron affinity (A), electronegativity (χ), chemical hardness (η), softness (e), and electrophilicity index (ω) calculated on the basis of the energy of HOMO and LUMO are summarized in Table 5. The data show that the chemical hardness of the complexes is lower than that of the ligand. The electrophilicity index (ω) provides information not only about the reactivity but also about the toxicity of the molecule. This indicator is related to the stabilization of energy when the system acquires an additional electrostatic charge from the environment and quantifies the global electrophilic force of the molecule [51]. The electrophilicity index for the copper complex is much higher than that of the free ligand (four times higher), while for the cobalt complex it is twice as high as obtained for the ligand.

### 3.5. Docking Studies

Comparing the average number of hydrogen bonds formed with the B-DNA structure, it was observed that the number of H-Bonds does not change significantly in the case of decarbazine metal complexes compared to ligands. 

**Figure 7:**
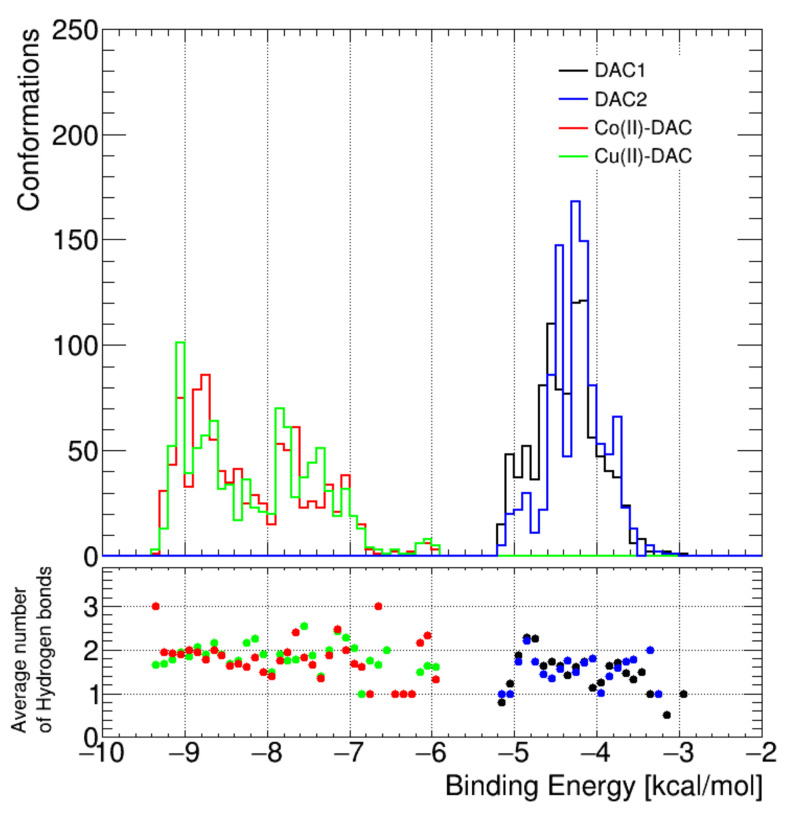
(**Upper**) Representation of the number of conformations formed by both ligand conformers (DAC1, DAC2) and metal complexes Co(II)-DAC and Cu(II)-DAC. (**Lower**) Average number of hydrogen bonds formed in complexes and ligands and free binding energy.

**Figure 8:**
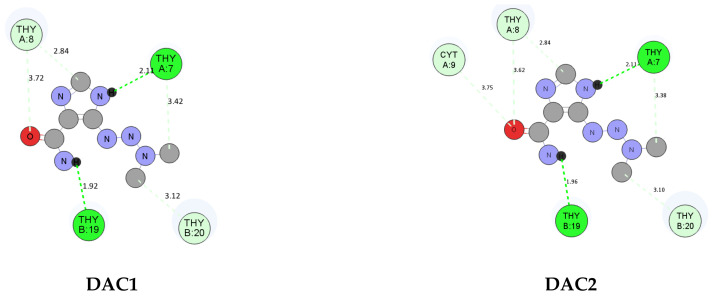

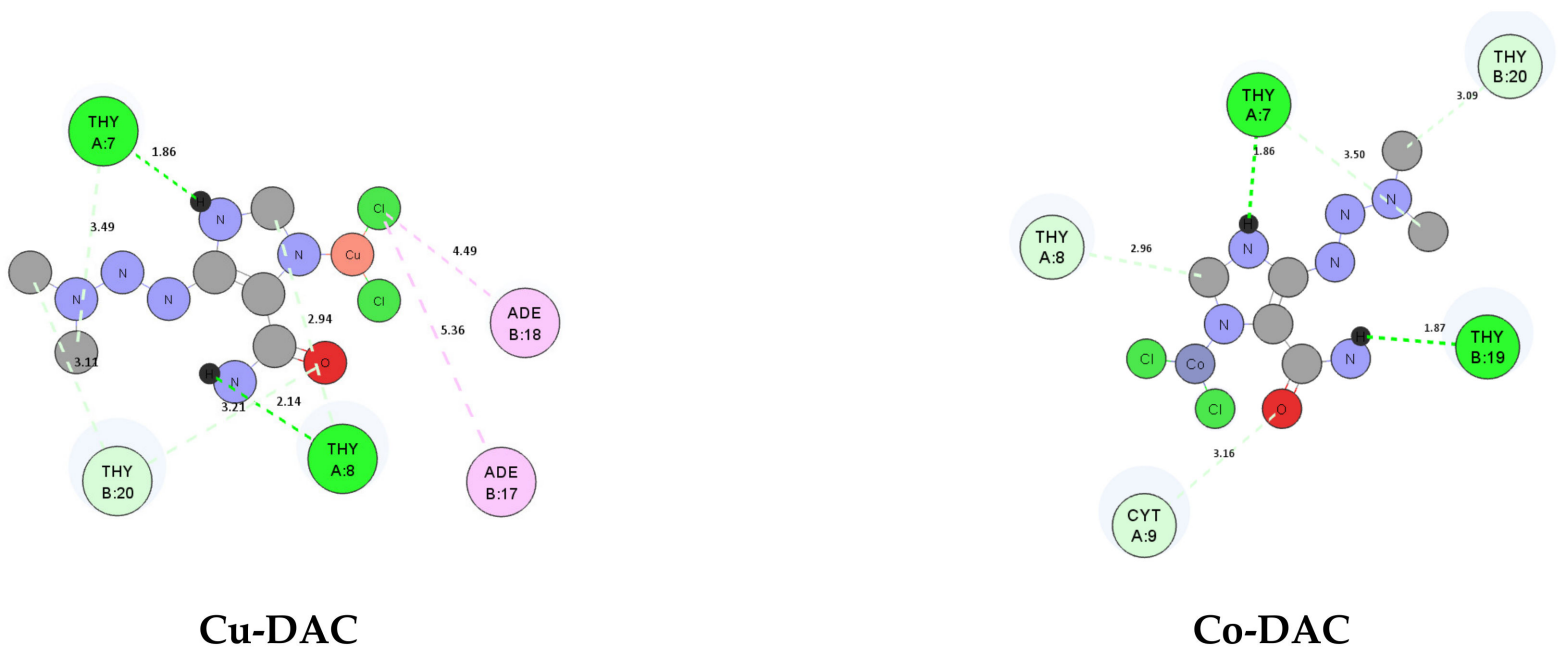

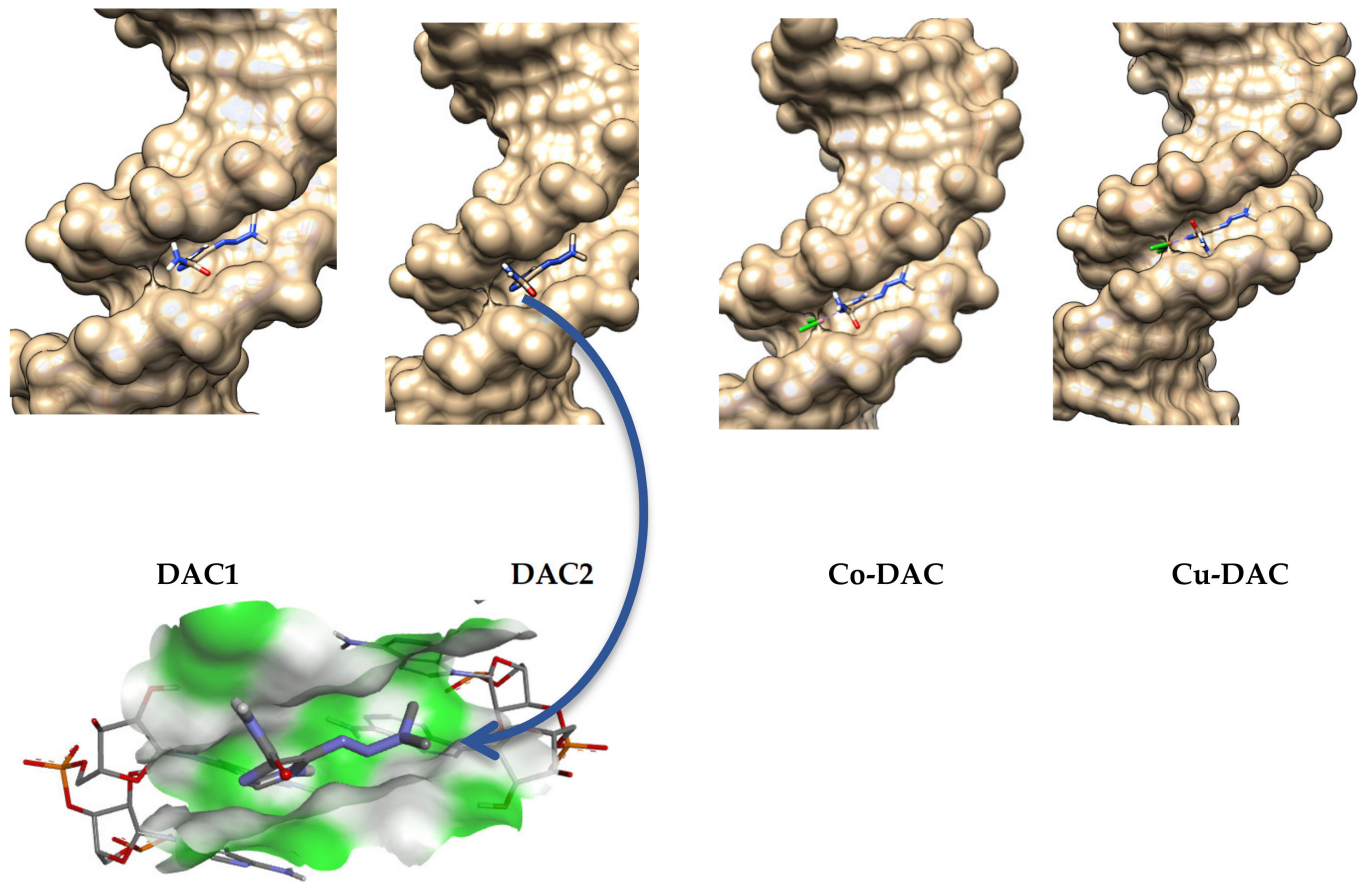
Best docking poses of decarbazine and complexes with B-DNA structure (**Upper**) in 2D with hydrogen bonds (green), Van der Waals (light green), and Pi-Alkyl (pink) interactions. (**Lower**) 3D visualization of location in the minor groove of DNA.

The obtained results indicate that the significant decrease (~4 kcal/M) in the free energy of binding the complexes with B-DNA is not due to formed hydrogen bonds (Figure 7). We conclude that formation of the complex causes favorable changes to occur in the distribution of the electronic charge. These changes in the electron density distribution affect the affinity to biological macromolecules like DNA. The tested compounds had an entropy value of S = 13.75 kcal/mol and an internal energy with values Dac1 (−4.37 kcal/mol), Dac2 (−4.26 kcal/mol), Cu-DAC (−8.13 kcal/mol), and Co-DAC (−8.19 kcal/mol), respectively (Table 6). The most energetically advantageous complexes with DNA formed two hydrogen bonds with thymine (Figure 8). In the case of constant inhibition, the value for metal complexes decreased by more than 1000-fold, and the free-binding energy decreased by about 4 kcal/mol.

In Ahmad and Ahmad’s studies, the binding constant (Kb) was 7.89 × 10^4^ experimental isothermal titration calorimetry of ct-DNA-DAC, and Kb = 6.99 × 10^4^ in docking studies for B-DNA-DAC [52]. Dacarbazine–ctDNA binding energy obtained from ITC experiments was –5.49 kcal/mol and it is comparable with minimized free energy of DAC–DNA obtained in docking studies (−5.35 kcal/mol). The most favorable interactions were found in the A-T residue of the minor DNA groove and hydrogen bonding and van der Waals interactions likely play a major role in these interactions. Previous research described by Wang et al. [53] obtained by atomic force microscopy and nanostructural image of DAC–DNA complexes indicate interaction at some specific site of the DNA sequence without intercalation. Additionally, DAC shows poor binding affinity to ssDNA [53]. Based on cyclic voltammetry, differential pulse voltammetry studies, and using spectroscopic methods performed by Radi et al., it has been concluded that decarbazine binds to double-stranded DNA by the combined effect of intercalation and electrostatic interactions [54].

Temerk and Ibrahim [19] describe that both DAC and DAC-Cu(II) complexes intercalate with base stacking of dsDNA, independent of ionic strength. In contrast, the interaction of a DAC with ssDNA, which is negatively charged, is electrostatic attractions. However, in the presence of Cu(II), these interactions are much stronger. Values of standard Gibbs free energy (−Δ*G◦*) in 278 K for DAC-ssDNA is 28.74 kJ/M (6.87 kcal/M) and for DAC-Cu(II)-ssDNA is 29.98 kJ/M (7.17 kcal/M) with enthalpy Δ*H◦* = 21.98 kJ/M and 22.29 kJ/M, respectively. For comparison −Δ*G◦* for DAC-dsDNA is 25.75 kJ/M and 27.55 kJ/mol for DAC-Cu-dsDNA [19].

The presented results indicate the formation of beneficial interactions and changes in the distribution of the electronic charge in decarbazine complexes with copper and cobalt, which in turn may enhance the interaction with macromolecules such as DNA.

### 3.6. Thermogravimetric Study

**Figure 9:**
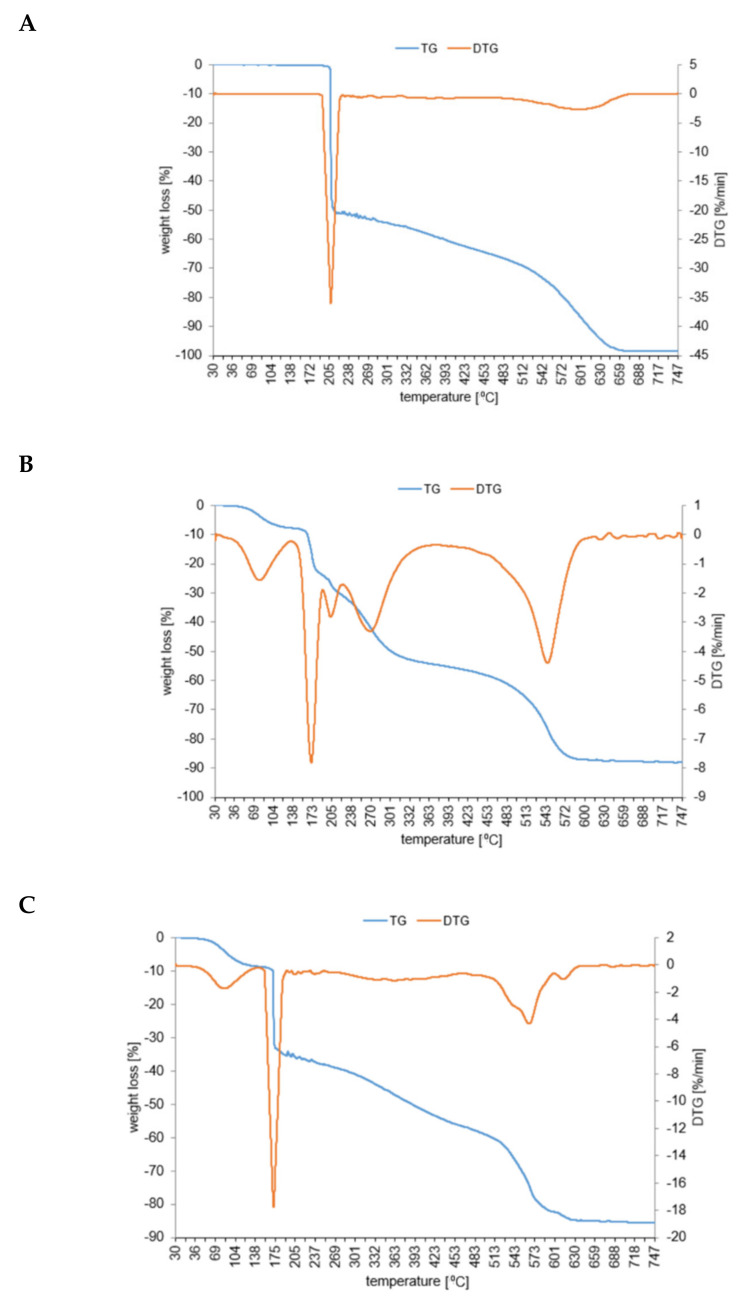
TG/DTG curves of dacarbazine (**A**), cobalt (**B**), and copper (**C**) complexes.

Thermogravimetric curves of dacarbazine and its metal complexes with copper(II) and cobalt(II) are shown in Figure 9. Dacarbazine underwent immediate decomposition above 205 °C. Rapid mass loss observed on the TG curve was accompanied by a narrow signal on the DTG curve. In the first stage of thermal decomposition of dacarbazine (in the range of 205–210 °C), a 50% loss in weight occurred. The mid weight loss was due to degradation of the triazene group and thermal dissociation of the amide group. The second stage of thermal decomposition of decarbazine was less rapid (above 201 °C)—the imidazole ring and C=O group totally decomposed, and the total mass loss was at 675 °C (Table 7). 

In the first stage of thermal decomposition of copper(II) and cobalt(II) complexes of dacarbazine the process of methanol desolvation occurred. The cobalt complex totally lost methanol (1.75 mole of CH3OH) in the range of 60–120 °C, and the copper complex (1.5 mole of CH3OH) in the range of 60–110 °C. The thermal decomposition of cobalt compound started at 175 °C (at a much lower temperature than decarbazine). The first stage of decomposition was as rapid as it was in the case of ligand (with a 33% loss in weight). The second stage of decomposition ran gently until achievement of 540 °C, and then the rapid mass loss occurred. The TG curve possessed a similar shape as the TG curve of decarbazine. At 630 °C the cobalt complex totally decomposed to CoO.

At the beginning of the thermal decomposition of copper(II) complex of dacarbazine the methanol desolvation occurred. Next the thermal decomposition of the complex started at about 160 °C (a slightly lower temperature than in the case of dacarbazine). During the thermal decomposition of copper complex, no stable intermediate products were observed (similarly as in the case of the cobalt complex). Above 580 °C the final product of decomposition CuO occurred. Comparing the curves of thermal decomposition of studied compounds it appeared that complexes of dacarbazine were less stable than ligand alone. The thermal decomposition of cobalt and copper complexes started at temperatures 35 and 50 degrees lower than ligand, respectively. The cobalt complex of dacarbazine was more thermal stable than the copper complex.

## 4. Conclusions

The spectroscopic IR and NMR studies revealed that dacarbazine coordinates metal cations (cobalt(II) and copper(II)) through the oxygen atom of carbonyl group and nitrogen atoms from the imidazole ring. It was stated on the basis of significant shifts of bands assigned to the vibrations of the aforementioned groups from the infrared spectra and chemical shifts of protons from the ^1^HNMR spectra (changes in the electronic charge density under the influence of complexation). The thermal analysis showed that cobalt(II) and copper(II) complexes of dacarbazine were less stable than ligand. The cobalt complex of dacarbazine was more thermal stable than the copper complex.

Based on the literature data (X-ray diffraction) and spectroscopic data, the theoretical structures of the complexes were modeled. Experimentally determined and theoretically calculated structures were compared. For the modeled structures, the distribution of electronic charge, aromaticity, and energies of the HOMO and LUMO orbitals were calculated. This allowed the assessment of the effect of complexation with copper and cobalt on the stabilization of the aromatic system and the reactivity of the investigated complexes. Complexation with copper and cobalt changes the electronic charge distribution of dacarbazine. The aromatic system of dacarbazine is stabilized (aromaticity indices increase) due to metal complexation. The values of the calculated energy of the HOMO and LUMO orbitals indicate an increase in the reactivity of the dacarbazine molecule after complexation. This has a significant impact on the biological activity of these substances. The electron charge distribution of a ligand provides information about the stability of a given compound as well as its reactivity and biological activity. By complexing ligands of high biological importance with metals, we influence the electronic stability of a given ligand and, at the same time, we can influence its biological activity. Additionally, by examining the thermal properties of given chemical compounds, we can potentially check their susceptibility to thermal utilization and test the decomposition products.

## Figures and Tables

**Table 1:** The wavenumbers from the IR (theoretical and experimental) and Raman spectra of dacarbazine and IR spectra of dacarbazine complexes with cobalt and copper.

Calculated IR (B3LYP 6-311G(d,p)						Experimental Spectra (cm^−1^)				
DAC 1			DAC 2			DAC		Cobalt Complex	Copper Complex	
Unscaled	Scaled = 0.967	Intensity	Unscaled	Scaled = 0.967	Intensity	IR	Raman	IR	IR	Assignments
3703	3581	105.50	3728	3605	72.84	3383 s	3371 w	3406 s	3406 s	ν_as_NH_2_
3647	3527	58.23	3560	3443	65.93	-	-	3322 s	-	νNH
3549	3432	60.67	3585	3467	53.44	3269 m	-	-	-	ν_s_NH_2_
3238	3131	2.26	3240	3133	2.09	3174 m	-	3188 s	3186 s	νCH_ar_
3144	3040	7.76	3143	3039	11.28	3147 m	3141 vw	-	-	ν_s_CH_3_
3139	3035	8.76	3139	3035	5.32	2964 m	-	-	-	ρ_s_CH
3085	2983	13.51	3097	2995	15.07	2905 m		2925 m	2923 m	ν_as_CH_3_
3078	2976	26.13	3074	2973	28.84	2793 m	-	-	2796 w	ν_as_CH_3_
3014	2915	47.62	3021	2921	40.93	2753 m	-	-	-	ν_s_CH_3_
3003	2904	78.48	2997	2898	94.76	2612 m	-	-	-	ν_s_CH_3_
1749	1691	494.07	1740	1683	326.18	1609 vs	1604 w	1605 s	1609 s	νC=O
-	-	-	-	-	-	1656 vs	-	1637 vs	1639 vs	νCH_3_
1620	1567	141.69	1607	1554	256.98	-	-	-	-	ρC-NH_2_
-	-	-	-	-	-	1561 w	1559 w	1561 m	1566 m	ρ_s_CH_3_
1569	1517	3.59	1586	1534	55.59	1510 m		1525 s	1528 s	νCN
1527	1477	197.90	1525	1475	155.81	1476 s	1488 vw	1487 vs	1488 vs	ρC-NH_2_
1483	1434	27.90	1481	1432	25.74	1436 m	1449 vs	1440 m	1440 m	ρNH_2_
1451	1403	75.22	1453	1405	32.35	1402 s	-	1405 m	1405 m	νCC, ν_ring_, ρNH_2_
1438	1391	5.80	1438	1391	9.68	-	-	-	-	νCC, αN-CH_2_, βNCH
1431	1384	47.98	1430	1383	74.13	1381 s	1388 w	-	-	β_as_CH_3_, ν_ring_
1389	1343	21.23	1388	1342	24.58	1344 s	-	1352 vs	1351 vs	β_s_CH_3_, ν_ring_
1363	1318	114.39	1367	1322	408.84	1304 m	-	1306 s	1307 s	β_s_CH_3_, ν_ring_
1331	1287	36.74	1328	1284	40.87	1270 w	1289 vw	-	-	β_s_CH_3_, ν_ring_, βNCH
1270	1228	2.30	1260	1218	16.53	1259 w	1258 vw	1253 vs	1255 m	νCN, βCH
1231	1190	67.15	1233	1192	20.71	1231 m	1204 vw	-	-	ν_ring_, βCH
1153	1115	25.50	1152	1114	39.12	1183 w	-	-	-	βCH + νNN
1136	1099	26.51	1137	1099	9.08	1072 s	1091 vw	1091 s	1091 s	νNN, βCH
1114	1077	24.06	1110	1073	7.62	-	-	-	-	βCH + βCNC
1091	1055	202.22	1093	1057	17.91	1049 w	1012 m	-	-	ρCH_2_, βCH
1084	1048	12.72	1075	1040	117.70	986 w	-	-	-	ρC-NH_2_, βCNC
1062	1027	14.22	1061	1026	18.40	962 w	-	969 m	980 w	ρCH_2_, βCNC
951	920	4.32	955	923	15.06	904 w	913 vw	902 w	902 w	ρCH_3_, βCH
915	885	14.86	912	882	16.25	896 w	-	-	-	ρCH_3_, ν_ring_
817	790	9.74	812	785	5.02	882 w	-	-	-	Δring, ν_ring_(CN)
799	773	2.21	799	773	2.40	868 w	873 vw	-	-	ρCH_3_, ν_ring_
797	771	10.95	798	772	14.79	796 w	-	-	-	βNCH, βNCC
672	650	13.44	683	660	14.27	690 w	691 w	-	-	βNH, βCCO
668	646	0.82	679	657	0.01	653 vw	-	-	-	β(CCN + NH)
632	611	25.62	602	582	43.51	630 m	-	646 m	649 m	αNNN, αCNC
586	567	0.50	596	576	0.65	542 m	-	565 m	568 m	ρNH_2_, αNNH, αCCO
552	534	73.21	574	555	104.33	450 w	445 vw	-	-	ring_def_
550	532	39.82	568	549	10.64	-	-	-	-	ring_def_
447	432	10.01	449	434	11.86	416 vw	-	-	-	αC-NH_2_

* Abbreviation: band intensity: s—strong, m—medium, w—weak, vw—very weak, type of vibrations: ν—stretching, ρ—bending out-of-plane β—bending in-plane, α—deformation in-plane, Δ—deformation out-of-plane, ring_def_—deformation of ring, s—symmetric, as—asymmetric.

**Table 2:** The chemical shifts (ppm) from the ^1^HNMR theoretical (GIAO/B3LYP/6-311++G**) and experimental spectra of dacarbazine and experimental spectra of cobalt and copper complexes with dacarbazine.

		DAC 1	DAC 2	Dacarbazine Complexes	
				Cobalt	Copper
H (amide group)	Exp.	7.29, 7.41	-	6.20, 5.40	-
	Theoret.	6.86, 4.57	6.87, 4.40	6.38	8.29, 5.42
H (CH_ring_)	Exp.	7.83	-	8.04	-
	Theoret.	7.20	6.98	8.90	7.74
H (NH_ring_)	Exp.	11.52	-	-	-
	Theoret.	8.56	8.72	9.43	8.81
H (CH_3_-triazene group)	Exp.	3.13, 3.50	-	3.49	-
	Theoret.	3.67, 3.48, 3.36, 3.19, 2.29, 2.59	4.73, 4.37, 3.91, 3.88, 3.69, 3.17	3.78, 3.74, 3.71, 3.44, 3.34, 3.14	3.62, 3.61, 3.61, 3.24, 3.23, 3.03

**Table 3:** Geometrical aromaticity parameters and selected bond lengths and angle values obtained for dacarbazine and copper(II) and cobalt(II) complexes of dacarbazine.

	Dacarbazine			Copper Complex		Cobalt Complex
	Exp [46]	DAC1	DAC2	Exp [47]	Calc	Calc
Energy	-	−638.54	−638.54	-	−3199.60	−2941.88
Dipole m	-	10.7498	5.5647	-	21.8411	22.5754
**Aromaticity indices**						
HOMA	0.905	0.893	0.880	0.925	0.897	0.890
GEO	0.060	0.069	0.079	0.057	0.065	0.067
EN	0.035	0.039	0.041	0.017	0.039	0.043
I5	67.97	67.39	65.35	70.44	68.42	67.68
**Bond lengths [A]**						
C1-O1	1.230	1.218	1.222	1.362	1.236	1.247
C1-N1	1.338	1.370	1.366	1.309	1.345	1.342
C1-C2	1.470	1.492	1.484	1.460	1.477	1.465
C2-C3	1.379	1.392	1.388	1.387	1.393	1.392
C3-N2	1.375	1.377	1.381	1.370	1.383	1.381
N2-C4	1.352	1.370	1.367	1.345	1.356	1.359
C4-N3	1.333	1.307	1.309	1.311	1.311	1.312
						
N3-C2	1.387	1.376	1.383	1.379	1.379	1.384
C3-N4	1.383	1.384	1.380	1.387	1.382	1.380
N4-N5	1.285	1.270	1.279	1.286	1.273	1.274
N5-N6	1.304	1.325	1.326	1.299	1.314	1.313
N6-C5	1.451	1.456	1.458	1.450	1.455	1.455
N6-C6	1.449	1.453	1.451	1.450	1.458	1.458
**Angles (°)**						
N2-C3-N4	127.50	125.36	125.07	116.37	115.48	116.15
C3-N4-N5	112.80	113.71	113.23	112.17	115.88	115.46
N4-N5-N6	113.42	115.20	114.26	114.66	114.71	114.64
N5-N6-C6	116.55	115.90	116.01	122.46	121.71	121.70
C5-N6-C6	120.96	120.43	120.46	120.31	120.82	120.76
C1-C2-N3	120.18	120.93	121.18	112.88	113.31	112.19
O1-C1-N1	123.28	122.53	123.17	121.95	122.79	122.19

**Table 4:** NBO atomic charges calculated for dacarbazine and copper(II) and cobalt(II) complexes of dacarbazine.

	DAC1	DAC2	Copper Complex	Cobalt Complex
O1	−0.605	−0.805	−0.607	−0.582
N1	−0.820	−0.627	−0.775	−0.764
C1	0.635	0.635	0.647	0.648
C2	0.021	0.013	0.008	−0.002
C3	0.283	0.307	0.314	0.321
N2	−0.555	−0.552	−0.514	−0.513
C4	0.219	0.220	0.279	0.276
N3	−0.444	−0.509	−0.518	−0.470
q_ring_	−0.476	−0.521	−0.431	−0.388
N4	−0.346	−0.296	−0.343	−0.343
N5	−0.034	−0.047	−0.032	−0.031
N6	−0.248	−0.246	−0.215	0.213
C5	−0.390	−0.392	−0.390	−0.390
C6	−0.349	−0.349	−0.349	−0.349

**Table 5:** The values of energy of HOMO and LUMO orbitals and other electronic parameters calculated for dacarbazine and copper(II) and cobalt(II) complexes of dacarbazine.

	DAC1	DAC2	Copper Complex	Cobalt Complex
HOMO	−7.8342	−7.8391	−4.7827	−7.1092
LUMO	−4.7772	−4.7851	−4.4066	−5.5198
Energy gap	3.057	3.054	0.3761	1.5894
Ionization potential	7.8342	7.8391	4.7827	7.1092
Electron affinity	4.7772	4.7851	4.4066	5.5198
Electronegativity	6.3057	6.3121	4.59465	6.3145
Chemical potential	−6.3057	−6.3121	−4.59465	−6.3145
Chemical hardness	1.5285	1.527	0.18805	0.7947
Chemical softness	0.327118	0.327439	2.658867	0.629168
Electrophilicity index	13.00682	13.04604	56.13084	25.08677

**Table 6:** Molecular docking analysis results.

	Free Energy of Binding (kcal/mol)	Inhibition Constant (µM)	Intermolecular Energy	Torsional Energy	Unbound Extended Energy	Interacting Residues (H-Bonds) and Distance [Å]	Reference RMSD
DAC 1	−5.17	163	−5.99	0.82	−0.38	N1-THY B:19 (1.92) N2-THY A:7 (2.11)	25.45
DAC 2	−5.13	173.91	−5.95	0.82	−0.37	N1-THY B:19 (1.96) N2:THY A:7 (2.11)	25.43
Cu-DAC	−9.31	0.15106	−10.40	+1.10	−0.63	N1-THY:A8 (2.19) N2-THY A:7 (1.86)	25.26
Co-DAC	−9.33	0.14391	−10.43	+1.10	−0.56	N2-THY A:7 (1.86) N1-THY B:19 (1.87)	26.06

**Table 7:** The results of thermal decomposition of dacarbazine and their complexes with cobalt and copper.

Compound	Range of Decomposition	Weight Loss (%)		Products of Decomposition
		Calcd.	Found	
Dacarbazine	205–210	47.70	48.50	Imidazole +C*
	210–660	100	100	-
CoCl_2_(Dac)_2_·75CH_3_OH	60–120 175–630	10.19 86.38	10.34 85.61	CoCl_2_(Dac)_2_ CoO
CuCl_2_(Dac)_2_·1.5CH_3_OH	60–110 160–580	8.78 85.40	8.39 87.16	CuCl_2_(Dac)_2_ CuO

C*—carbon residue.

## Data Availability

The data presented in this study are available on request from the corresponding author.

## Supplementary Materials

The following are available online at https://www.mdpi.com/article/10.3390/ma14123274/s1, Figure S1. ^1^HNMR spectra for dacarbazine and dacarbazine complex with cobalt (II).
