# Supplementary figures and images for: Synthesis, Spectroscopic, and Theoretical Study of Copper and Cobalt Complexes with Dacarbazine
Source: Materials (Basel). 2021 Jun 13;14(12):3274. doi: 10.3390/ma14123274 (PMC8231934; doi:10.3390/ma14123274)

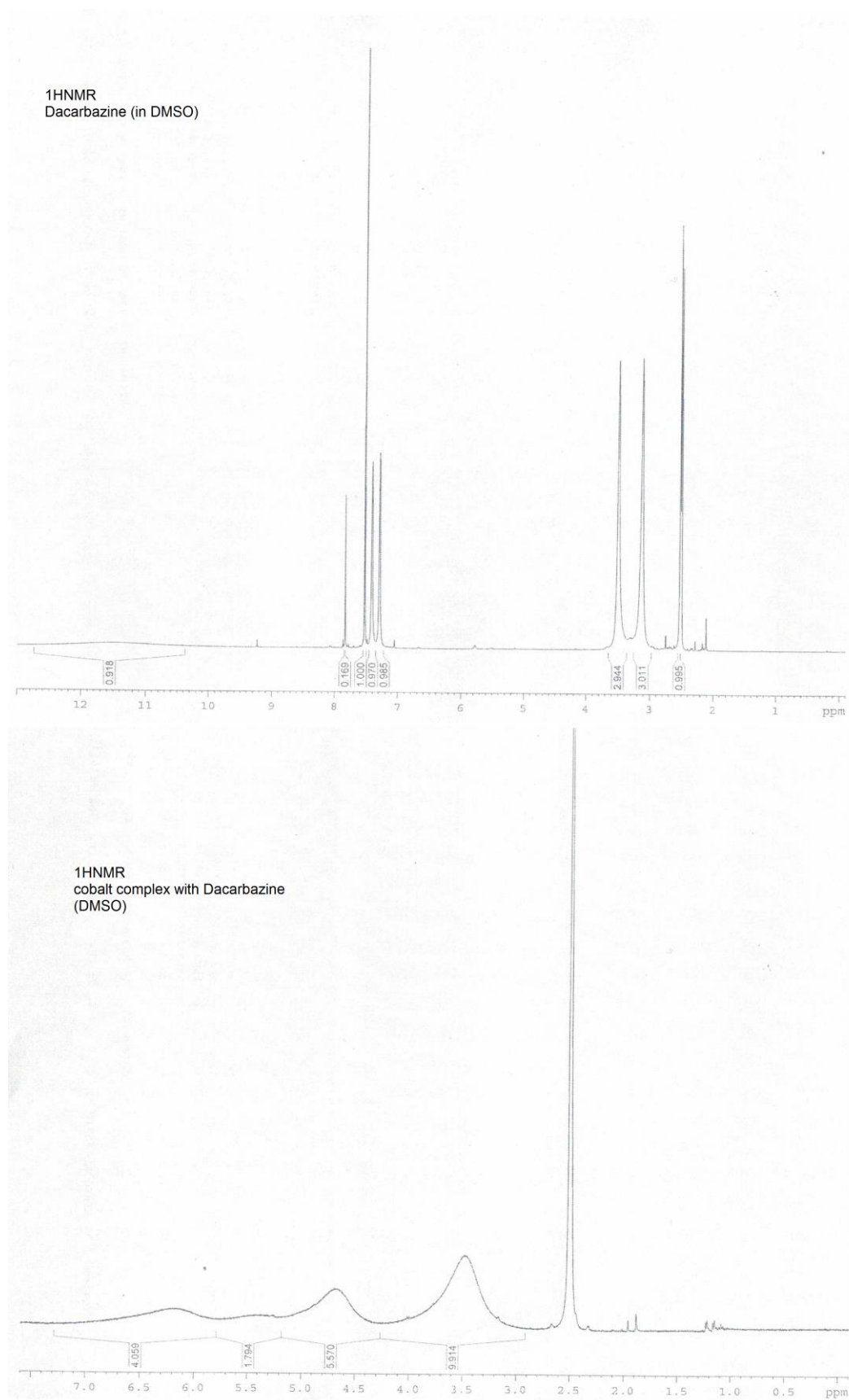

**Figure S1.** <sup>1</sup>HNMR spectra for dacarbazine and dacarbazine complex with cobalt (II).

Supplement: Supplementary file 1 [file materials-14-03274-s001.zip › materials-1206350-supplementary.pdf]
